# The Zurkurnai ECG Pattern: A Novel ECG Pattern of the High-Risk Features of Acute Pulmonary Embolism

**DOI:** 10.7759/cureus.52889

**Published:** 2024-01-24

**Authors:** Mohd Khairi Othman, Zurkurnai Yusof, Zul Khairul Azwadi Ismail, Khairil Amir Sayuti, W Yus Haniff W Isa

**Affiliations:** 1 Department of Internal Medicine, School of Medical Sciences, Universiti Sains Malaysia, Kubang Kerian, MYS; 2 Department of Radiology, School of Medical Sciences, Universiti Sains Malaysia, Kubang Kerian, MYS

**Keywords:** mc connels sign, pseudo wellen, right ventricular dysfunction, zurkurnai ecg pattern, acute pulmonary embolism

## Abstract

Acute pulmonary embolism is an important differential diagnosis in patients presenting with acute shortness of breath. However, the overlapping clinical presentation between acute coronary syndrome, aortic dissection, pneumonia, and heart failure made the diagnosis of pulmonary embolism very challenging in a limited resources center. We present a case of acute pulmonary embolism with an uncommon ECG pattern that was initially misdiagnosed as acute coronary syndrome.

The authors made the appropriate diagnosis using the Zurkurnai ECG pattern in acute pulmonary embolism, which is defined as the presence of right axis deviation, deep symmetrical T wave inversion in V1 to V5, II, III, and AVF with the maximum at V3-V4 and poor R wave progression, which indicates the high-risk features of acute pulmonary embolism.

## Introduction

Acute pulmonary embolism is an emergency condition, and early recognition is important, as it brings high mortality. Computed tomography pulmonary angiography (CTPA) is a gold-standard investigation tool for diagnosing acute pulmonary embolism; however, it is not widely available in all centers. Hence, a high index suspicion of acute pulmonary is important when encountering a patient with sudden onset dyspnea. The most easily available tool is electrocardiography (ECG). Multiple literature reviews have described changes in ECG in acute pulmonary embolism; however, it has low specificity. ECG changes, such as sinus tachycardia, S1Q3T3, and right strain pattern, have been described, and they show a correlation with acute pulmonary embolism despite low sensitivity [1-5]. Biphasic T wave inversion in the precordial lead has been described as Wellens’ pattern ECG and correlates with coronary artery occlusion rather than acute pulmonary embolism [6,7]. To date, biphasic T inversion at anterior and inferior lead are uncommon presentations of acute pulmonary embolism [6]. Hence, we describe Zurkurnai’s ECG pattern as one of the ECGs of the high-risk features of acute pulmonary embolism. The Zurkurnai ECG pattern is defined as the presence of right axis deviation, deep symmetrical T wave inversion in V1 to V5, II, III, and AVF with the maximum at V3-V4 and poor R wave progression, which indicates the high-risk features of acute pulmonary embolism.

## Case presentation

**Figure 1:**
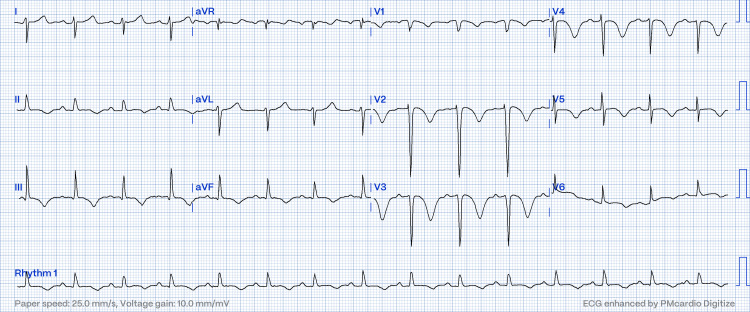
ECG showed the Zurkurnai ECG pattern defined as right axis deviation, poor R wave progression, biphasic deep T inversion on V2-V5 and limb leads, maximum at V3

A 43-year-old man with no known traditional coronary artery disease risk factors and a previous history of pulmonary embolism in 2022 due to long-distance travel completed T. Rivaroxaban 15 mg BD for three months. He presented with acute shortness of breath with exertion, which worsened over three days. It was associated with palpitation. There were no other associated symptoms. Upon arrival at the emergency department, his blood pressure was 118/87 mmHg, and his pulse rate was 107 bpm with 100% saturation under room air. Other clinical examinations revealed no significant findings. Initial blood investigations showed total leucocytes 7.1x109/L, hemoglobin 17.2 g/dL, and platelets 372x109/L. His coagulation profile showed activated partial thromboplastin time (aPTT) of 34.30 secs, prothrombin time (PT) of 14.70 secs, and international normalized ratio (INR) of 1.08. His troponin T level was 26 (normal range <15pg/L). Other blood investigations were within the normal range. ECG showed sinus rhythm, right axis deviation, poor R wave progression, and deep symmetrical T inversion at V2-V4 (Figure 1). There was also the presence of minimal T inversion in leads II, III, and AVF as compared to the precordial lead.

**Figure 2:**
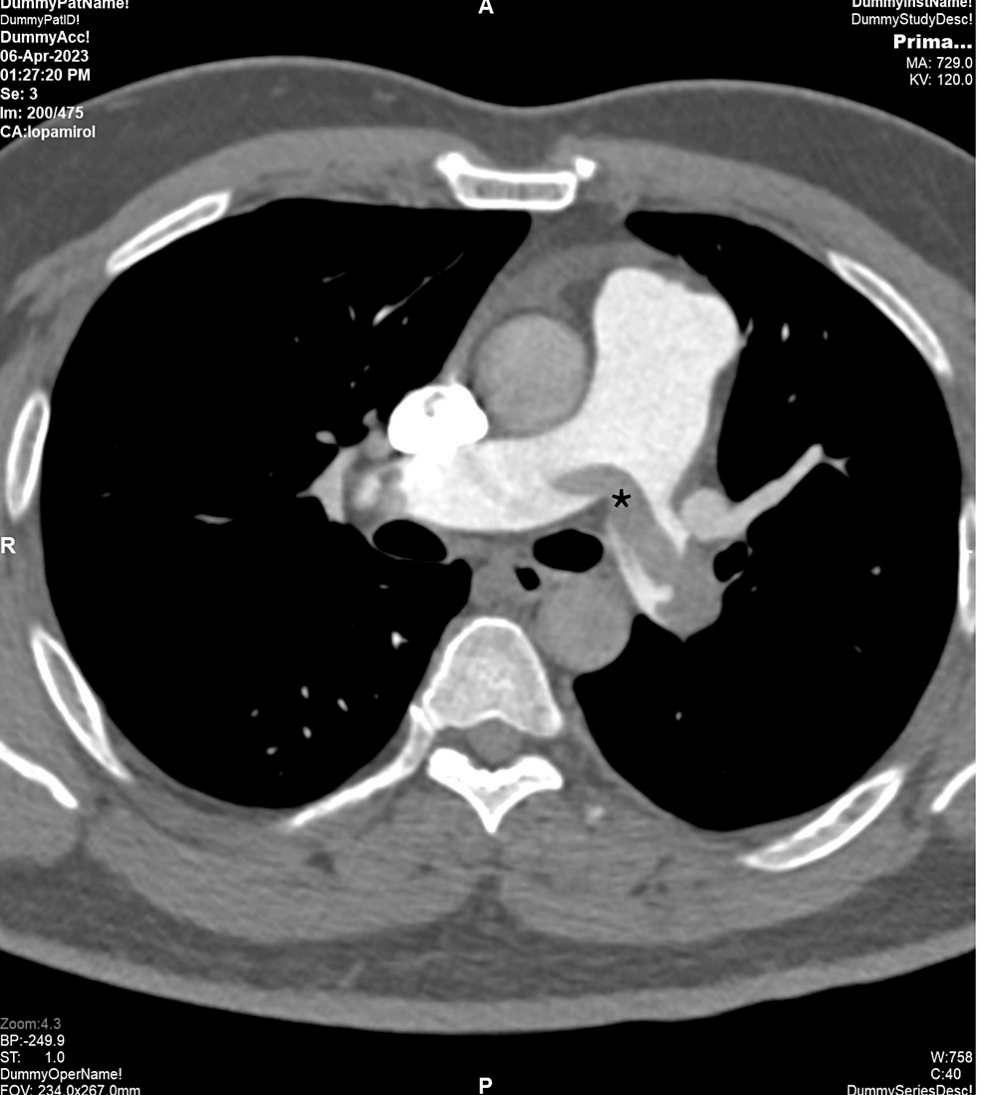
Axial CPTA at the level of the main pulmonary artery bifurcation shadowing a large filling defect (labeled with an asterisk) straddling the main pulmonary artery extending to the right and left pulmonary arteries consistent with saddle embolism Note the dilation of the pulmonary artery relative to the corresponding ascending aorta. CPTA: CT pulmonary angiography

**Figure 3:**
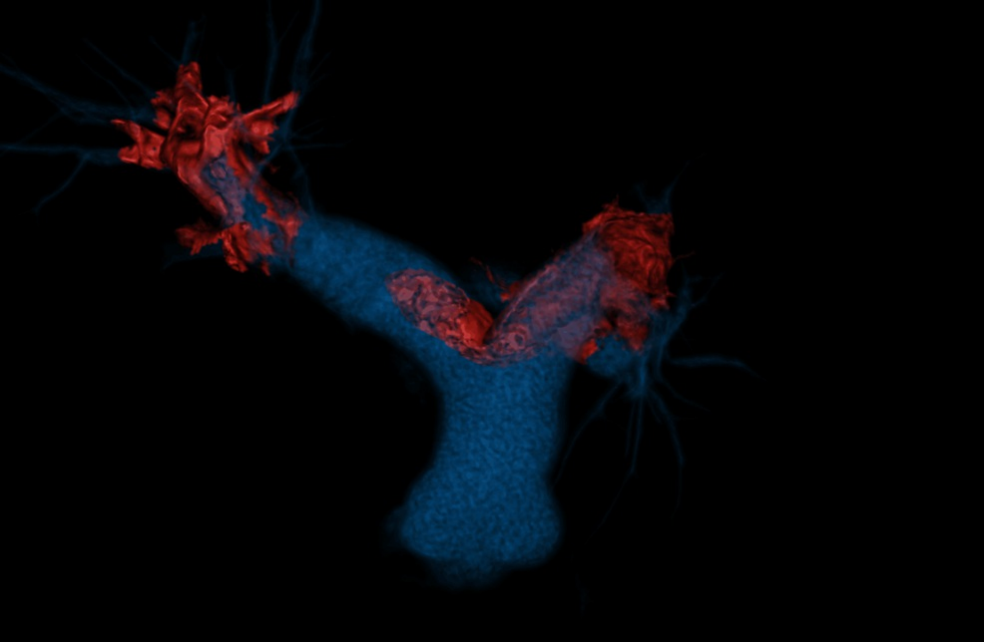
VRT reconstruction of the pulmonary artery and its proximal branches showing extensive pulmonary embolism (in red) involving the main pulmonary artery, right and left pulmonary arteries, and proximal segmental branches Note the dilation of the pulmonary arteries consistent with pulmonary hypertension. VRT: volume rendering technique

The chest X-ray was grossly normal. His initial diagnosis was non-sT elevation myocardial infarction (NSTEMI), and he was admitted to the hospital by the general medical team. Dual antiplatelet and anticoagulant were initiated. Echocardiography was performed the next day and showed dilated right ventricle (RV), right ventricle fractional area changes (FAC) of 29%, McConnell’s sign, and mobile thrombus at main pulmonary artery bifurcation (Videos 1-3). Computed tomography pulmonary angiography confirmed the presence of saddle pulmonary thrombus at the bifurcation of the pulmonary trunk extending to the left pulmonary artery until subsegmental branches (Figure 2 and Figure 3). His CT coronary angiography showed normal coronaries.

**Video 1:** Transthoracic echocardiography of the short axis view showed a D-shaped left ventricle with a dilated right ventricle size secondary to pulmonary hypertension

**Video 2:** Transthoracic apical 4 chamber view showed the presence of right ventricular free wall akinesia and hyperkinetic of the RV apex suggestive of McConnell's sign, which is specific to acute pulmonary embolism

**Video 3:** Transthoracic echocardiography of the RV inflow view showed the presence of mobile thrombus at the main pulmonary artery bifurcation

He subsequently received thrombolysis with intravenous streptokinase 250,000 units for 30 minutes, followed by 100,000 units over 24 hours after cardiology team consult. He was discharged well from the ward after one week of hospitalization with T. Rivaroxaban 15 mg BD for three months, and unfortunately, he did not turn up for follow-up at three months.

## Discussion

Acute pulmonary embolism is a life-threatening condition if the diagnosis is delayed. The clinical symptoms and initial ECG manifestations are variable, making the diagnosis of pulmonary embolism a challenging one. Increased right ventricular pressure will lead to right ventricular dilation, tricuspid regurgitation, and right ventricular dysfunction. Furthermore, there will be a loss of interventricular dependence due to right ventricle pressure overload [8]. These pathophysiological changes may lead to surface ECG manifestation. In our patient, there is the presence of right ventricular dysfunction and increased RV pressure as consequences of acute pulmonary embolism.

The diagnosis of acute pulmonary embolism based on ECG is difficult, as it has been described as sinus tachycardia, right axis deviation, S1Q3T3, right bundle branch block, T wave inversion in right precordial leads, P pulmonale, or atrial flutter/fibrillation, which are clues for the diagnosis of acute pulmonary embolism; however, it has varied sensitivity and specificity [1,4,9]. The presence of right ventricular hypertrophy (RVH) is one of the diagnoses of pulmonary embolism [1]. RVH, as defined by Sokolow-Lyon criteria, which is a composite of amplitude (R voltage of V1 + S voltage of V5 or V6) more than 10.5 mm was defined, was absent in this patient [1]. Right axis deviation is one of the classic manifestations associated with acute pulmonary embolism. A right ventricular strain pattern is described as the presence of RVH with ST-segment depression and T wave inversion, mainly in leads V1 and V2. In precordial leads, leads V3 or V4 are often referred to as ‘transition zones’ due to the magnitude of R and S waves becoming equivalent [3]. A shift of transition zone in acute pulmonary embolism has been described [3]. It has been postulated that biochemical abnormalities and hypoxia of the myocardium and His-Purkinje system cause slowing conduction in the right ventricle. Studies have shown that ST-segment and T-wave changes are associated with acute pulmonary embolism [3,5]. Our patient’s T-wave inversion in V1 to V5 and lead II, III, and AVF concur with previous reports. The new findings in our patient, which differ from previous reports, were the presence of poor R progression, deep symmetrical T-wave inversion in V1 to V5, and maximum at V3. The most likely explanation is severely dilated RV, leading to ischemic changes mimicking the proximal left anterior descending artery occlusion. The ECG findings in this patient have not been described in previous reports.

Apart from the ECG, transthoracic echocardiography is helpful for the early identification of echocardiography. A literature review of transthoracic echocardiography has described important findings that may suggest acute pulmonary embolism [10,11]. The echocardiographic finding is important as acute pulmonary embolism, myocardial infarction, or aortic dissection may have similar clinical presentation. In acute pulmonary embolism, there is an increased pressure inside the RV, which can be seen in transthoracic echocardiography. Based on the European Society of Cardiology guideline in 2019, transthoracic echocardiographic findings such as enlarged ventricle in long parasternal axis view, dilated RV with basal ratio RV/LV >1, presence of McConnel signs, flattened intraventricular septum, dilated inferior vena cava, 60/60 sign, decreased tricuspid annular plane systolic excursion (TAPSE), and decreased peak systolic (S’) velocity of tricuspid annulus should be looked into when assessing patient suspected acute pulmonary embolism [10]. However, the echocardiographic findings showed a low negative predictive value; none can rule out pulmonary embolism, especially in significant acute pulmonary embolism without significant RV hemodynamic effect. In our patient, the diagnosis of acute pulmonary embolism was made given the presence of mobile right heart thrombus in which an uncommon finding and addition of echocardiographic of RV dysfunction, which makes acute pulmonary embolism the diagnosis. It is an uncommon finding of the presence of a mobile right heart thrombus, and it is associated with a high mortality rate of approximately 44% [11].

CTPA is a gold standard for diagnosis of acute pulmonary embolism. CTPA has a high sensitivity and specificity based PIOPED II trial demonstrating 83% and 96%, respectively. Besides the diagnosis, CTPA can also give a clue of risk features of acute pulmonary embolism such as backwash contrast, abnormal septal morphology, dilated pulmonary trunk, and RV/LV ratio of more than 0.9 in the short-axis view [12,13]. Recent literature reported that a centrally located embolism is associated with a two-fold increase in mortality compared to distal thrombosis [14]. 

In combination with echocardiography and CTPA findings, it can be concluded that our patient had high-risk features of acute pulmonary embolism.

## Conclusions

Acute pulmonary embolism is a condition that contributes to high mortality if the diagnosis is late. A high index of suspicion should be maintained when encountering a patient with a previous history of venous thromboembolism presenting with acute coronary symptoms. The combination of the Zurkurnai ECG pattern and echocardiography plays a vital step in diagnosing this condition promptly.
